# Supplementary material for: The Dutch Pregnancy Drug Register: Suitable to Study Paternal Drug Exposures?
Source: Int J Environ Res Public Health. 2023 Nov 24;20(23):7107. doi: 10.3390/ijerph20237107 (PMC10706075; doi:10.3390/ijerph20237107)
Supplement: Supplementary file 1 [file ijerph-20-07107-s001.zip › ijerph-2658923-supplementary.pdf]

International Journal of
Environmental Research and Public Health

MDPI

Table S1. Corresponding ATC codes for the drug classes.

L04AA24	L04AB04	L04AA34	D11AH04	L04AA32	L04AX01	L04AA37	L04AC02	L04AA28	L04AA26
L04AC12	L04AC08	L04AB05	P01BA01	L04AD01	L01AA01	D11AH05	L04AB01	L04AA18	L04AA45
L04AB06	P01BA02	L04AB02	L04AC13	L04AA13	L01BB02	A07EC02	L04AX03	L04AA06	L04AA06
A07EC03	D11AH02	L04AC18	L01XC02	L01EJ01	L04AC14	L04AC10	L04AA10	A07EC01	L04AD02
D11AH01	L04AC17	L01BB03	L04AC07	L04AA29	L04AA44	L04AC05	L04AA33		

Table S2. Time to pregnancy in immunosuppressant group and control group.

	Immunosuppressant group	Control group	*Crude P* value	*Adjusted P value**
**Time to pregnancy**			0.55	0.66
≤1 year	77 (86.5%)	7966 (84.2%)		
>1 year	12 (13.5%)	1493 (15.8%)		
*Unplanned pregnancy***	*3*	*823*		
*Unknown***	*0*	*55*		

* Adjusted for: maternal and paternal age, maternal and paternal country of birth, maternal and paternal education level of both father, maternal BMI, and gravidity. **Not included in the analysis

Table S3. Fertility treatments performed in order to conceive in immunosuppressant group and control group.

	Immunosuppressant group	Control group	*P* value
**Fertility treatment**			0.84
No	81 (91.0%)	8582 (90.4)	
Yes	8 (9.0%)	915 (9.6%)	
*Unplanned pregnancy**	*3*	*823*	
*Unknown**	*0*	*17*	
**Reduced fertility of the men**			0.43
No	5 (62.5%)	677 (74.5%)	
Yes	3 (37.5%)	232 (25.5%)	
*Unknown**	*0*	*6*	

*Not included in the analysis.
